# Correction: Disrupting the transmembrane domain–mediated oligomerization of protein tyrosine phosphatase receptor J inhibits EGFR-driven cancer cell phenotypes

**DOI:** 10.1016/j.jbc.2022.102416

**Published:** 2022-09-05

**Authors:** Elizabeth Bloch, Eden L. Sikorski, David Pontoriero, Evan K. Day, Bryan W. Berger, Matthew J. Lazzara, Damien Thévenin

It was determined via STR profiling that cell line UMSCC2 should have been labeled as HSC3 cells. Like UMSCC2 cells, HSC3 cells are squamous cell carcinoma cells that express wild type EGFR, the primary substrate of PTPRJ/DEP1 investigated in this publication. Therefore, this correction does not affect the results and conclusions of the paper.

